# Muscle Activation and Ground Reaction Force between Single-Leg Drop Landing and Jump Landing among Young Females during Weight-Acceptance Phase

**DOI:** 10.3390/sports11090185

**Published:** 2023-09-18

**Authors:** Metaneeya Pilanthananond, Kittichai Tharawadeepimuk, Vitoon Saengsirisuwan, Weerawat Limroongreungrat

**Affiliations:** 1 Department of Physiology, Faculty of Science, Mahidol University, Bangkok 10400, Thailand; metaneeya.pil@mahidol.edu (M.P.); vitoon.sae@mahidol.ac.th (V.S.); 2 College of Sports Science and Technology, Mahidol University, Nakhon Pathom 73170, Thailand; kittichai.tha@mahidol.ac.th

**Keywords:** lower limb, single-leg landing, neuromuscular control, female, EMG

## Abstract

Single-leg drop landing (SLDL) and jump landing (SLJL) are frequently used as assessment tools for identifying potential high-risk movement patterns; thus, understanding differences in neuromuscular responses between these types of landings is essential. This study aimed to compare lower extremity neuromuscular responses between the SLDL and SLJL. Thirteen female participants performed an SLDL and SLJL from a 30-cm box height. Vertical ground reaction force (vGRF), time to peak vGRF, and surface electromyography (sEMG) data were collected. Continuous neuromuscular responses, peak vGRF, and time to peak vGRF were compared between the tasks. Statistical parametric mapping (SPM) analysis demonstrated that the SLJL had a significantly higher sEMG activity in the rectus femoris (RF), vastus lateralis (VL) and vastus medialis (VM) within the first 10% of the landing phase compared with SLDL. At 20–30% of the landing phase, sEMGs in the RF and VL during the SLDL were significantly higher compared with SLJL (*p* < 0.05). A higher peak vGRF and shorter time to peak vGRF was observed during SLJL (*p* < 0.05). In conclusion, our findings highlight that SLJL exhibited greater RF, VL, and VM activities than SLDL at initial impact (10% landing), coinciding with a higher peak vGRF and shorter time to attain peak vGRF. Our findings support the role of the quadriceps as the primary energy dissipator during the SLJL.

## 1. Introduction

Single-leg landings are extensively used to evaluate the movement pattern of the lower extremities and screen for lower limb injury risk (i.e., anterior cruciate ligament (ACL) and ankle sprain) [1]. Despite different types of landings being employed in biomechanical studies, the most common types have been drop jump and jump landings [2,3,4]. The single-leg drop landing (SLDL) represents a functional sporting task [2] that can be studied in isolated conditions, with the landing height able to be predetermined and controlled within laboratory settings. While the SLDL serves as a common model for investigating landing mechanics [3,4], fall mechanics [5], and landing-associated injury mechanisms [6], drop landing movements may not fully represent sport-specific actions. This has led to the single-leg jump landing (SLJL) being utilized as an additional landing condition or an alternative test to mimic sports scenarios better [1,7].

The adoption of drop jumps is based on the assumption that (1) landing mechanics are similar to jumping when landing from an identical height [5,8], and (2) the velocity of the center of mass (CoM) is the same when landing from a jump [5]. However, past studies comparing a bilateral drop jump against a countermovement jump (CMJ) using a matched CoM fall height have demonstrated drop jump landings to possess a greater vertical ground reaction force (vGRF) and a more rapid time to peak vGRF [5,9]. During the weight acceptance phase of a CMJ landing, the subsequent take-off phase is facilitated by lowering the CoM, resulting in a different fall height from the same box height [5], thus impacting landing biomechanics. The aforementioned is an example of how coordination strategies are adjusted according to the constraints of the task and its potential risk to the system [10,11]. In regards to an SLJL, the necessity for the CoM to move forward (prior to landing) represents diversity in task criteria that can potentially affect landing biomechanics [11], which is not well documented.

Using surface electromyography (sEMG), Afifi and Hinrichs [5] also compared muscle activation during bilateral jump landings. A higher quadriceps, lateral hamstring, and calf muscle activity were noted after initial contact with the CMJ compared with the drop jump [5]. This was proposed to be due, in part, to the greater joint flexion angles in the CMJ task [5] and the eccentric contraction of the quadriceps opposing the landing-initiated external knee flexion to prevent the collapse of the lower extremities [5,12,13]. In contrast to bilateral jump assessments, there is a lack of studies that have attempted to compare muscle activity between single-leg landing tasks. In addition, the omission of tracking muscle activity over the entirety of the landing phase has not enabled temporal differences in muscle activation to be determined. However, this limitation may be overcome via the application of statistical parametric mapping (SPM) to sEMG time-series data [14]. The adoption of the SPM approach offers the advantage of avoiding data over-simplification from discretization-induced bias [15,16,17]. The dependency between adjacent values is considered rather than separately performing inferential tests at each time point [18]. This results in a more comprehensive analysis of rapid changes in muscle activation during the landing phase.

While it is important to assess landing mechanics across both genders, female participants are more susceptible to developing a landing injury [4,19,20,21] with an increased valgus collapse, knee abduction, and greater vGRF during drop jump landings [4,22]. Moreover, females are reported to have higher rectus femoris activity when landing compared to males [4,23]. In combination, these characteristics have been identified as predictive factors of ACL injury in many sports activities [4,24,25]. Nevertheless, neuromuscular responses during sport-specific jump landing movements, such as the SLJL, where task constraints are different from more controlled movements (i.e., SLDL or bilateral landings), remain to be fully elucidated.

To our knowledge, a comparison between the SLJL and SLDL lower extremity neuromuscular responses across a landing phase is yet to be comprehensively studied. Understanding the biomechanical requirements and neuromuscular responses of the lower leg muscles across the landing phase of SLDL and SLJL may assist in clarifying the selection of the most suitable test to fulfill specific study objectives. Accordingly, this study aimed to compare leg muscle activation and vGRF between the SLDL and SLJL during the weight acceptance phase using a female participant cohort. Using SPM, we hypothesized that the SLJL would exhibit higher quadriceps muscle activation and an accompanying greater vGRF and faster force application when compared to the SLDL.

## 2. Materials and Methods

### 2.1. Participants

Thirteen healthy females (age = 24.6 ± 4.2 years, height = 158.0 ± 7.0 cm, body mass = 53.4 ± 8.2 kg, body mass index (BMI) = 21.4 ± 2.4 kg·m^2^) volunteered to participate in this study. Participant inclusion criteria included (1) occasionally involved in any kind of sport and/or physical activity (≤3 h of exercise per week), (2) Q-angle within 10–20°, (3) leg length discrepancy not exceeding 2 cm [26] and (4) BMI within 18.5–25 kg·m^2^. The exclusion criteria included: (1) knee joint deformity, (2) feet supination or over-pronation, (3) pelvic side shift, (4) recurrent or recent injuries of the lower extremities, (5) musculoskeletal problem on either leg within the past three months, and (6) history of neurological disease, vestibular, or visual disturbance. The physical activity readiness questionnaire (PAR-Q) and the participant’s medical history were also evaluated prior to participation. All participants were informed about the study’s purpose, procedures, and potential risks before providing their written informed consent to participate. The minimum number of required participants was determined based on a pilot study using 5 participants, during which peak sEMG activity of the rectus femoris (RF) was obtained during an SLDL and SLJL. Using G*Power software 3.1.9.7 [27], an ɑ priori power analysis for a two-tailed paired sample *t*-test indicated that a sample size of 12 participants would be necessary to reliably detect an effect size (*d*) of 0.89 with 80% power at a significance level (ɑ) of 0.05. All procedures were conducted in compliance with the 1964 Declaration of Helsinki and its later amendments and were approved by the University Institutional Review Board (MU-CIRB 2020/077.2503). The study is registered in the Thai Clinical Trials Registry (TCTR20220129003).

### 2.2. Experimental Design

Participants visited the biomechanics laboratory on two separate occasions. During the first visit, they were familiarized with all test procedures, including practicing SLDL and SLJL movements. The strong leg of each participant (not necessarily the dominant leg) was determined [28] using a horizontal hop for distance while barefoot with hands on hips. This task was completed twice for both legs, and the leg that yielded the greater average hop distance was defined as the strong leg. A stretching warm-up was employed prior to the hop tests. On the second visit, participants changed into spandex shorts and a t-shirt. Surface electrodes (Ambu Blue Sensor P; P-OO-S, Ballerup, Denmark) were affixed to skin sites that had been shaved, abraded, and cleaned with alcohol. The electrodes were positioned on muscle bellies in accordance with surface electromyography for the non-invasive assessment of muscles (SENIAM) guidelines [29] and separated by an interelectrode space of 1.5 cm. The electrodes were taped to the skin and wrapped with elastic bandages around the limb to limit movement artifacts. To ensure the quality of each jump, we put a retroreflective marker on the participants’ sacrum (S2), a proxy for representing the center of mass (CoM). The trajectory of the S2 marker was traced in real-time with a 3-D optoelectronic motion analysis system (BTS Bioenginering Inc., Garbagnate Milanese (MI), Italy). The successful forward jump was identified when the vertical position of the marker during the flight phase did not exceed the position of the participant when standing atop the box.

### 2.3. Single-Leg Landing Tasks

**Figure 1:**
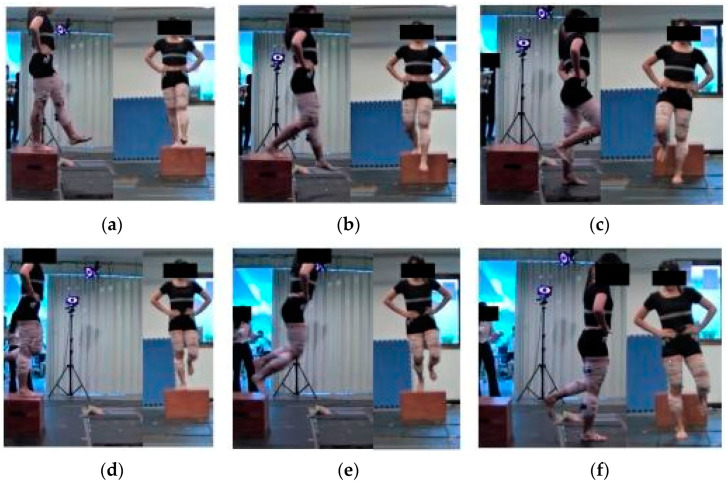
Two landing tasks implemented in this study. (**a**–**c**) SLDL test; (**d**–**f**) SLJL test.

In a randomized, counterbalanced order, participants performed either an SLDL or SLJL while standing barefoot atop a 30 cm box before landing on the center of a force plate using their strong legs. The 30-cm box and 70-cm jump distance have been employed as standard task criteria in a number of landing studies [30]. In the SLDL trial, the box was placed 30 cm away from the center of the force plate. Upon a verbal signal, participants leaned forward and dropped onto the force plate while maintaining their balance. In the SLJL trial, the box was placed 70 cm from the center of the force platform [1,30]. Participants stood on the box with their strong legs and jumped forward to land on the force plate with the same leg while maintaining their balance (Figure 1). Participants were instructed to avoid upward jumping, and the S2 marker trajectory was monitored in real time to ensure this was the case.

In both tasks, participants were encouraged to fully extend their landing leg upon successful balance recovery. Participants kept their hands on their hips to limit upper limb movement during each jump performance. Three to five familiarizations with a 3–5-min recovery were permitted before proceeding to the collected trials to minimize injury risk and reinforce the correct landing form. Each participant completed three successful experimental trials for each landing condition with a 3-min recovery between trials. Unsuccessful trials, where participants were unable to regain balance or dropped their non-landing leg to the ground, were repeated. All data were collected from the period when participants stood atop the box to the moment of full leg extension upon landing. The EMG data of the landing leg was recorded from the following sites: rectus femoris (RF), vastus lateralis (VL), vastus medialis (VM), bicep femoris (BF), semitendinosus (ST), tibialis anterior (TA), medial gastrocnemius (MG), and peroneus longus (PL). Peak vGRF and time to peak vGRF were calculated using MATLAB 2020b software (The MathWorks, Inc., Natick, MA, USA).

### 2.4. Data Processing

The EMG data collected from initial contact (vGRF threshold > 20 N) to the lowest center of mass were used for analysis. The GRF data underwent filtering using a fourth-order Butterworth low-pass filter at 20 Hz, while the raw sEMG data were band-pass filtered within the range of 15–500 Hz. Subsequently, the raw sEMG data were processed using the root-mean-square (RMS) method with a mobile window of 20 ms [31]. All EMG data were amplitude normalized to its peak value and time normalized to 100 data points. Additionally, the data were ensemble averaged across three trials for each participant. Peak vGRFs were normalized with each participant’s body weight and then averaged across the three trials for each task.

### 2.5. Statistical Analysis

**Figure 2:**
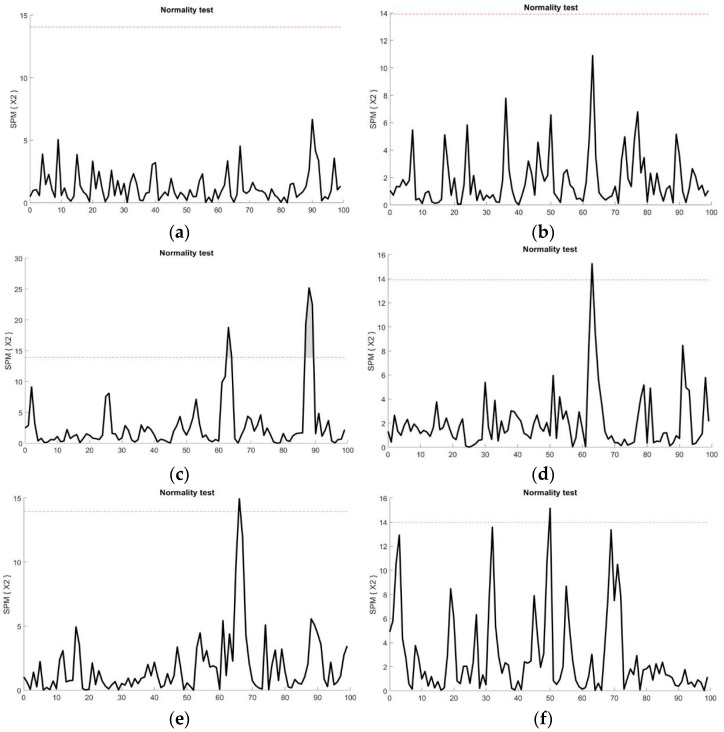

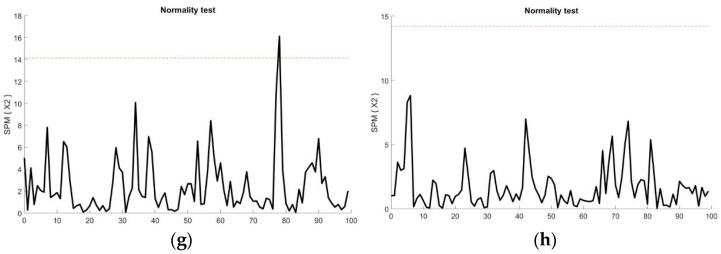
Shapiro Wilk test for normality. EMG times-series of (**a**) RF, (**b**) VL, and (**h**) PL were below the critical threshold (dashed line), demonstrating a normal distribution. However, some parts of the EMG times series of (**c**) VM, (**d**) BF, (**e**) ST, (**f**) TA, and (**g**) MG were above the critical threshold, demonstrating a non-normal distribution.

The sEMG times-series data of each muscle, from initial contact to the end of the landing phase, was tested for a normal distribution using a Shapiro-Wilk test (Figure 2). For the analysis of normally distributed sEMG data, we utilized SPM analysis, and for non-normally distributed data, we used statistical non-parametric mapping (SnPM). In particular, the covariance between the EMG time series was elucidated using the SPM Hotelling’s $T^{2}$ statistic, which is a vector-field equivalent to univariate tests such as the two-sample *t*-test [32,33]. The sEMG data during the weight-acceptance phase were analyzed as a vector field *I* = 8, *J* = 13, *Q* = 101. The calculation was applied and retrieved from Robinson, Vanrenterghem, and Pataky [34] as follows:

(1) $$SPM\{T^{2}\}=T^{2}(Q)=\frac{J_{1}J_{2}}{J_{1}+J_{2}}{\left(\bar{y_{1}}(q)-\bar{y_{2}}(q)\right)}^{T}W{\left(q\right)}^{-1}(\bar{y_{1}}(q)-\bar{y_{2}}(q))\;\;$$

(2) $$W=\frac{1}{J_{1}+J_{2}-2}\;(\sum_{j=1}^{J_{1}}(y_{1j}-\bar{y_{1}})({y_{1j}-\bar{y_{1}})}^{T}+\sum_{j=1}^{J_{2}}(y_{2j}-\bar{y_{2}})({y_{2j}-\bar{y_{2}})}^{T}\;)$$

where *I* was the number of vector components, *J* was the number of participants’ responses, *Q* was the number of time points, *q* was the calculation at each individual time point, and *W* was the pooled covariance matrix, respectively. Subscripts “1” and “2” indexed the two groups. The significant difference in sEMG time series between tasks was identified when the $SPM\{T^{2}\}$ values exceeded the critical threshold, which was further analyzed using post-hoc analysis. This test hierarchy was comparable with the post-hoc *t*-tests following ANOVA. The comparison of each individual vector component $(y_{i}\left(q\right)$) may be conducted when overall significance is achieved in the vector field (y(q)) analysis [34]. In this study, a paired *t*-test was employed to examine any differences in muscle sEMG time-series data between the two landing tasks. Significant differences in sEMG amplitudes within the time-series data were defined when the time-varying *SPM*{*t*} or *SnPM*{*t*} values exceeded a critical threshold (t*).

The peak vGRF and time-to-peak vGRF data were also tested for a normal distribution using the Shapiro-Wilk test. If the data were normally distributed, we analyzed condition differences in peak vGRF and time to peak vGRF using a paired sample *t*-test. Alternatively, if the data were non-normally distributed, a Wilcoxon signed-rank test was employed. All data are reported as mean standard deviation (SD) unless otherwise stated. Statistical significance was accepted at the *p* < 0.05 level. MATLAB 2020b software (The MathWorks, Inc., Natick, MA, USA) was used for statistical analysis.

## 3. Results

### 3.1. Muscle Activation

Analysis of sEMG amplitude time-series data across the landing phase showed that sEMG activity in the RF, VL, and PL muscles fitted a normal distribution (Figure 2a,b,h). In contrast, sEMG data for the VM, BF, ST, TA, and MG muscles were found to fall above the t* during the landing phase, indicating non-normally distributed data (Figure 2c–g).

**Figure 3:**
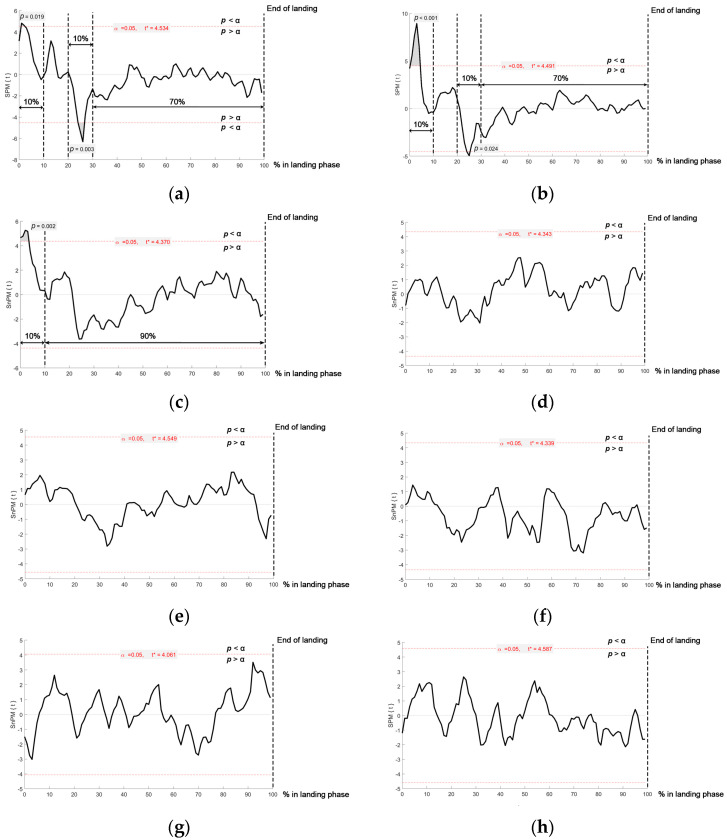
SPM and SnPM analysis (paired *t*-test) demonstrating the comparison of (**a**) RF, (**b**) VL, (**c**) VM, (**d**) BF, (**e**) ST, (**f**) TA, (**g**) MG, and (**h**) PL times-series activation between SLDL and SLJL. Red dashed lines demonstrate a critical threshold. A significant difference in muscle activation between tasks is shown when SPM{t} or SnPM{t} values exceeded the threshold (*p* < 0.05).

The paired sample *t*-test demonstrated that during the first 10% of the landing phase, there was a significantly higher sEMG activity in the RF (t* = 4.534; *p* = 0.019; Figure 3a), VL (t* = 4.491; *p* < 0.001, Figure 3b), and VM (t* = 4.370; *p* < 0.01) muscles in the SLJL compared with SLDL. Conversely, during 20–30% of the SLJL landing phase, sEMG activity was significantly lower in the RF (t* = −4.534; *p* < 0.01; Figure 3a) and VL (t* = −4.491; *p* < 0.024, Figure 3b) muscles compared with the SLDL. There were no significant differences in sEMG activity observed in the other muscles between jump tasks across the same period during the landing phase (all *p* > 0.05; Figure 3d–h). In addition, no significant difference in sEMG muscle activity was noted between movements during the remainder of the landing phase.

### 3.2. Peak vGRF and Time to Peak vGRF

The participants’ average peak vGRF and time to peak vGRF during both landing tasks are displayed in Table 1. Average peak vGRF and time to peak vGRF were significantly different between the SLDL and SLJL (*p* < 0.05). All participants demonstrated higher peak vGRF in the SLJL compared with SLDL. A shorter average time to peak vGRF was also exhibited in the SLJL compared with the SLDL during landing (Table 1).

**Figure 4:**
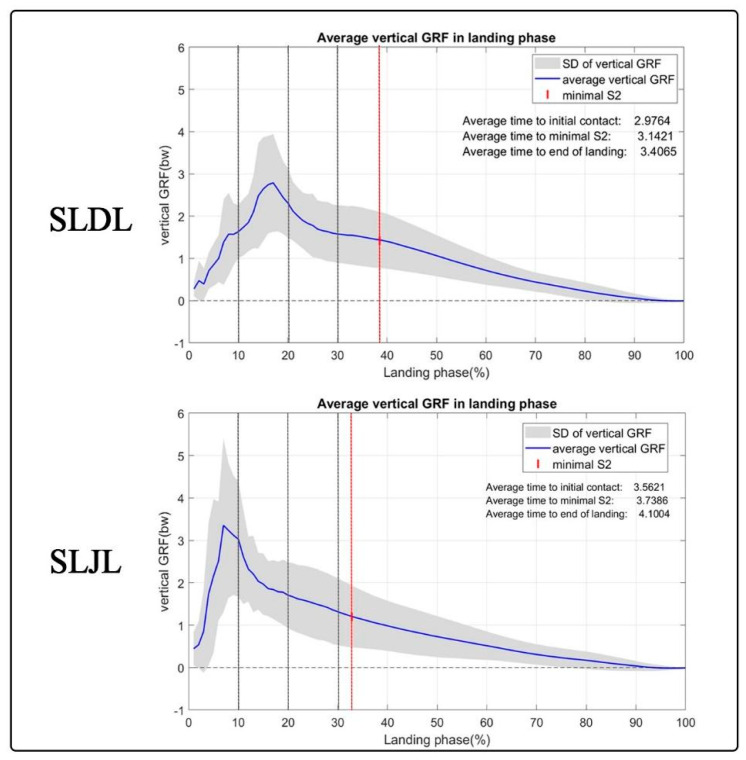
Average (blue line) ± SD (grey shade) vGRF in relation to the percent landing phase during the SLDL and SLJL. The red vertical line demonstrates the percent in the landing phase where the lowest vertical position of the S2 marker was achieved.

**Figure 5:**
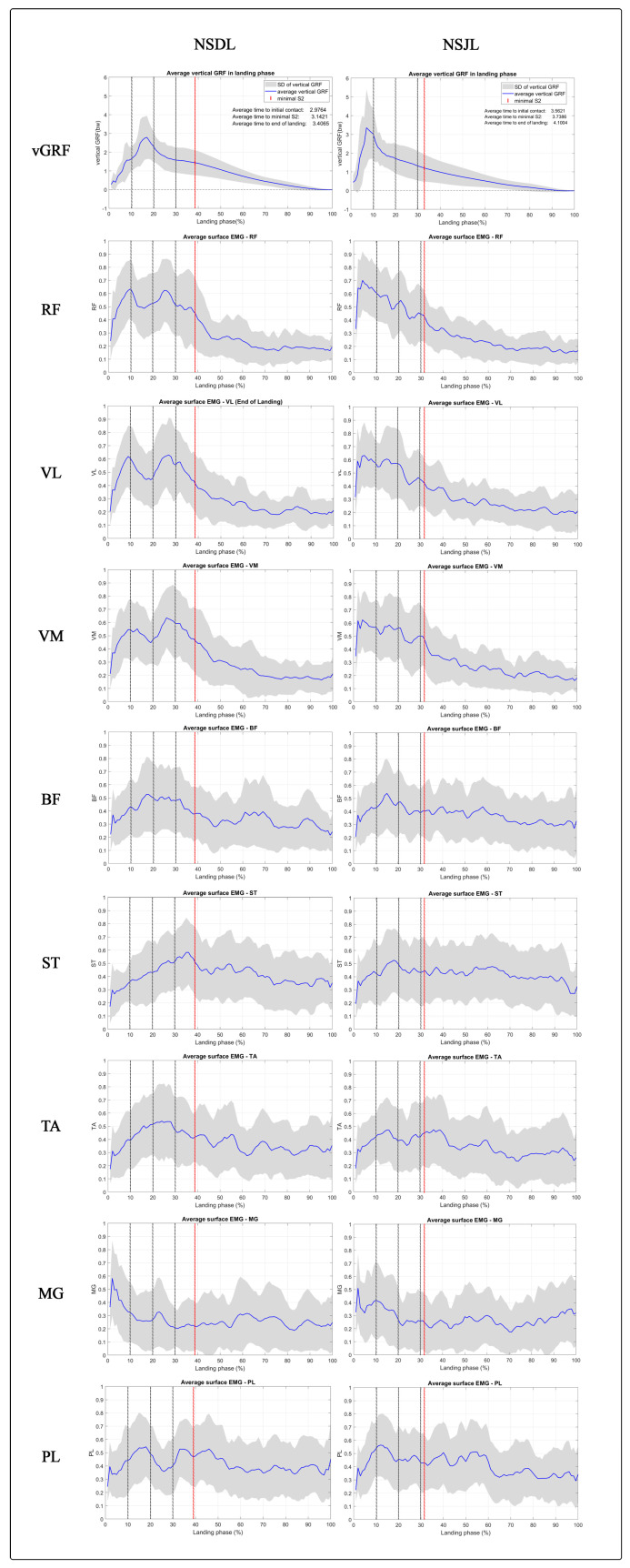
vGRF developed during SLDL and SLJL (first row) and average (blue line) ± SD (grey shades) sEMG signals of RF, VL, VM, BF, ST, TA, MG, and PL across the landing phase. The percent landing phase where the lowest vertical position of the sacral marker (S2) was achieved is marked with a red vertical line.

The vGRF in SLDL increased from initial contact and reached its peak within 10–20% of the landing phase before declining (Figure 4). In contrast, the peak vGRF developed during SLJL occurred within the first 10% of the landing phase (Figure 4). Figure 5 illustrates all sEMGs signals and vGRFs in relation to the percent landing phase during the SLDL and SLJL.

## 4. Discussion

The findings from this study confirmed our initial hypothesis that a higher EMG muscle activity would be observed in the quadriceps muscles, specifically in the RF, VL, and VM, during the initial 10% of the SLJL landing phase compared to the SLDL. In contrast, a higher sEMG activity was observed in the SLDL in the RF and VL muscles during 20–30% of the landing phase. There were no marked differences in hamstring or lower leg muscle activity between the two tasks. In addition to time-dependent differences in muscle activity, a higher peak vGRF and a shorter time to peak vGRF were observed in the SLJL condition, which also confirmed the initial hypothesis. Our findings demonstrate the distinct muscle activation patterns and force production strategies employed by female participants during SLJL and SLDL, contributing to a more in-depth understanding of the underlying biomechanical and neuromuscular mechanisms involved in these movements.

The higher sEMG activity observed in the RF, VL, and VM muscles during the initial 10% of the SLJL indicates a greater involvement of the quadriceps during the early landing phase of this movement compared with the SLDL (Figure 5). This greater activation of the quadriceps muscles during the initial part of the landing phase can be attributed to minimizing the lowering of the CoM (i.e., fall height) to maintain stabilization during the SLJL. Our present findings partly support this notion, as the S2 marker reached its lowest value earlier during the landing phase in the SLJL task (Figure 4). It can also be speculated that the higher accelerative force generated from jumping forwards with the SLJL and the associated higher peak vGRF and rapid time to peak vGRF (Figure 4) led to a higher eccentric quadriceps muscle activation after initial contact. Indeed, the quadriceps are understood to be the primary knee stabilizers during dynamic tasks, with the early eccentric contraction of the quadriceps decelerating joint flexion upon initial contact during the SLJL [3]. The quadriceps effectively help to absorb peak vGRF [5] and prevent force dispersion into bones and ligaments [1], potentially minimizing landing injury.

The SPM analysis revealed distinct patterns of quadriceps activation during the landing phase in both the SLDL and SLJL conditions. In the SLDL task, a second peak of quadriceps activation was noted at 20–30% of the landing phase, leading to markedly higher sEMG values in the RF and VL muscles than observed in the SLJL task (Figure 5). This second peak in quadriceps activation indicates a responsive action to further adjust the lower extremity position to absorb the landing impact force [35]. Nevertheless, despite the higher quadriceps muscle activation in the SLDL task at 20–30% of the landing phase, there was no difference in peak vGRF between conditions, and vGRF continued to decrease during this period (Figure 4).

In both tasks, high activity in RF, VL, and VM muscles continued to be noted during the first 50% of the landing phase (Figure 5), highlighting the importance of quadriceps activation during the early landing period in both movements. Conversely, hamstring sEMG activity was similar between jump tasks, with a tendency for higher muscle activity in the BF and ST muscles during the first 50% of the landing phase, especially in the SLDL condition. Additionally, there was a tendency for higher MG muscle activity following initial contact. Taken together, our findings are consistent with the work by Morgan et al. [36], who reported substantial forces generated by the quadriceps, gastrocnemii, and hamstrings during the weight-acceptance phase. In contrast to the quadriceps and hamstring muscles, the TA and PL demonstrated sustained activation during the initial 50% of the landing phase in both tasks, maintaining a relatively constant muscle activity throughout the entire landing phase (Figure 5). This activation in these lower leg muscles not only reveals the continual demand for muscle activations to achieve dynamic joint stabilization, maintain balance, and absorb landing energy throughout the entirety of the landing phase, but it could also provide a better understanding of muscle synergy surrounding both ankle and knee joints while performing the landing tasks.

Previously, other studies have relied upon discrete analysis to demonstrate different levels of lower limb activity between jump landing conditions using average sEMG values [37,38]. However, this approach has limitations in identifying the specific time points where differences occur within the landing phase. Discrete analysis tends to oversimplify the continuous data, introducing discretization-induced bias [15,16,17], which may result in misinterpretation. In contrast, SPM analysis, as employed in our study, overcomes these limitations by reducing bias to provide more detailed inferences about muscle activation comparisons between the SLDL and SLJL tasks.

The greater horizontal landing distance in the SLJL (70 cm) compared with the SLDL (30 cm) resulted in greater acceleration and a higher peak vGRF during the landing phase. The longer time to peak vGRF in SLDL reflects a lower biomechanical demand for this specific landing task. Our results contrast with a previous study by Afifi et al. [5], who demonstrated a shorter time to peak vGRF with a bilateral drop landing task. The discrepancy in time to peak vGRF is likely due to the different jump movements employed in both our studies. In our present study, the shorter time to peak vGRF in the SLJL indicates a reduced timeframe for landing energy dissipation or absorption by the muscles [39]. This implies that muscles in SLJL experience higher stress during a briefer period, having potential implications as an injury mechanism.

We focused solely on investigating the neuromuscular responses in a healthy female population due to females being at greater risk of sustaining a landing-related injury [4,19,20,21]. However, it is important to acknowledge that biomechanical demands and neuromuscular responses vary between genders due to hormonal, genetic, and, as described earlier, anatomical differences, such as dynamic knee angles in the frontal plane [4,24,25,40,41]. Male participants are reported to possess an increased quadriceps moment with increasing knee flexion during single-leg landings [42,43]. This coincides with males having a lower vGRF, suggesting different levels of quadriceps activation between males and females to dissipate the peak vGRF [4]. In addition, the difference in activation level and patterns among other lower extremity muscles contributes to vertical support, i.e., the knee extensors, soleus, and gastrocnemius [43]. Therefore, future work may consider studying other participant cohorts, such as male participants and individuals with specific conditions, for example, anterior cruciate ligament (ACL) injury or chronic ankle instability (CAI). Additionally, since our study only included healthy non-sport females, incorporating sportswomen in future studies is necessary in order to extend our findings to sports populations.

In the present study, we compared the activation of single muscles independently. However, as muscles are generally grouped and co-activated, the comparison of an inter-muscular coordination pattern (muscle synergy) during movement might be worth exploring in future studies. Furthermore, research varying the box height to and/or the horizontal landing distance relative to each participant’s jumping ability or the percentage of each participant’s body height [44,45] may be conducted.

Other limitations in our study include not purposively placing emphasis on tracking CoM or recording lower-limb kinematic variables using motion analysis software. This would have extended the scope of our work to explain potential between-task differences in drop height via tracking CoM alongside the accelerative forces acting upon the body upon landing. While not necessarily a limitation, as we specifically placed emphasis on neuromuscular responses during the landing phase, the addition of a vertical jump after landing would account for between-jump differences in the contribution of the stretch reflex response. As the end of musculotendinous stretching is associated with the end of the braking phase, investigating how the muscle stretch reflex contributes to the muscle activity during both tasks can provide a better understanding in terms of both muscle activation and mechanics (i.e., immediately after the post-landing phase).

## 5. Conclusions

The quadriceps muscles, specifically the RF, VL, and VM, showed higher sEMG activity during the initial 10% of the SLJL phase compared to the SLDL. Conversely, the RF and VL exhibited higher sEMG activity during 20–30% of the SLDL phase. In addition, a higher peak vGRF and a shorter time to peak vGRF were observed during the SLJL task, emphasizing the role of knee extensors as a force dissipator in SLJ, particularly at the initial impact (10% landing phase). These findings highlight the distinct neuromuscular responses and biomechanical demands associated with each movement. Consequently, the SLJL may be a more suitable test for representing sports-specific movements in laboratory settings, offering a closer simulation of real-world scenarios. Researchers should consider incorporating the SLJL as an alternative test to better replicate sports-related activities and enhance the ecological validity of studies.

## Figures and Tables

**Table 1:** Average peak vGRF and time to peak vGRF during the SLJL and SLDL.

	SLJL	SLDL	*p*-Value	Effect Size (*d*)
Peak vGRF (times BW)	4.89 ± 1.51	2.73 ± 0.44 *	0.001	−0.88
Time to peak vGRF (ms)	30.38 ± 9.81	48.15 ± 9.74 *	0.000	1.82

* Significant difference between two groups (*p* < 0.05).

## Data Availability

The data presented in this study are available on reasonable request from the corresponding author.
